# *Babesia microti* Infection, Eastern Pennsylvania, USA

**DOI:** 10.3201/eid1907.121593

**Published:** 2013-07

**Authors:** Marcela E. Perez Acosta, Peter T. Ender, Erin M. Smith, Jeffrey A. Jahre

**Affiliations:** St. Luke’s University Hospital and Health Network, Bethlehem, Pennsylvania, USA

**Keywords:** Babesiosis, Babesia microti, tick-borne illness, emerging disease, parasites, protozoa, vector-borne diseases, ticks, Pennsylvania, United States

## Abstract

Infection with *Babesia microti* has not been well-described in eastern Pennsylvania, USA, despite the vector of this organism being prevalent. We report 3 cases of babesiosis in eastern Pennsylvania in persons without recent travel outside the region or history of blood transfusions, suggesting emergence of this infection.

Babesiosis is an intraerythrocytic infection caused by protozoan parasites of the genus *Babesia.* In the United States, *Babesia microti* is the most common species that causes human babesiosis. Disease-endemic areas include specific regions in the northeast and upper Midwest United States. Infection with this organism can be asymptomatic to life-threatening. Signs and symptoms include high fever, diaphoresis, chills, headaches, and anorexia. Patients can also have hemolytic anemia and thrombocytopenia (*1*).

*B. microti* is transmitted by the *Ixodes scapularis* tick, which is also the vector of *Borrelia burgdorferi* and *Anaplasma phagocytophilum* (*2*). Although *B. burgdorferi* is endemic to Pennsylvania (*3*), *B. microti* is not considered endemic to this region (*4*,*5*). We report 3 cases of human babesiosis in patients from Northampton County in eastern Pennsylvania, USA, who had not recently traveled outside the region or had blood transfusions. None of the patients had risk factors for severe babesiosis, such as asplenia.

## The Patients

Patient 1 was a 68-year-old man who was hospitalized on August 18, 2011, because of 6 days of fever, arthralgias, generalized weakness, and confusion. He was given doxycycline for treatment of presumptive Lyme disease but showed no improvement. He had not traveled outside eastern Pennsylvania for >3 years. He had never received any blood transfusions. Although he did not recall any tick attachments, he enjoyed gardening and other outdoor activities.

Pertinent clinical and laboratory data are shown in the Table. During testing for thrombocytopenia, a peripheral blood smear was obtained on hospital day 1 and showed ring forms with tetrads (Figure) and a parasitemia level of 10%. A PCR result for the blood sample was positive for *B. microti*.

**Figure:**
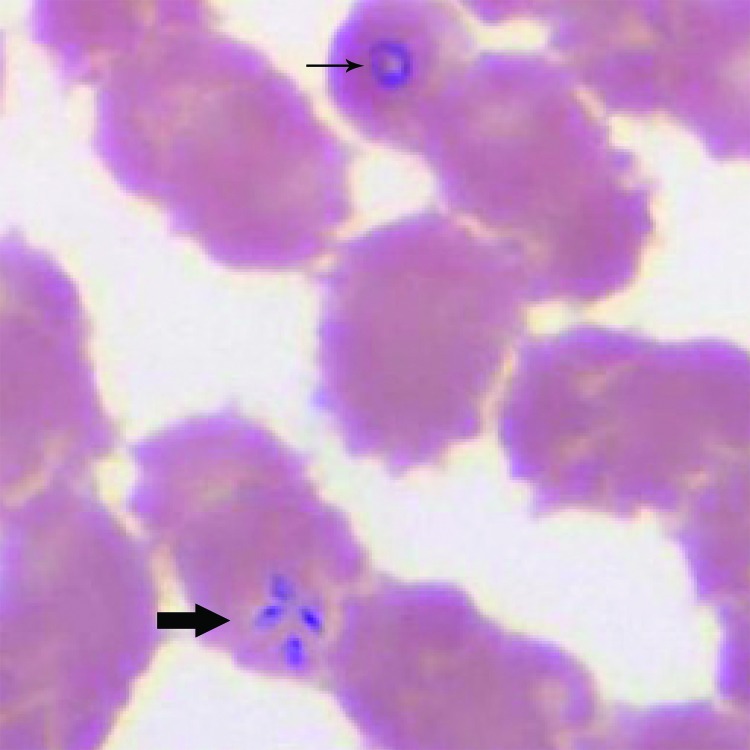
Wright-stained blood smear for patient 1 with babeosis on day 1 of hospitalization, eastern Pennsylvania, USA, showing an intraerythrocytic trophozoite of *Babesia microti* in a ring form (thin arrow) and a tetrad arranged in a cross-like pattern (thick arrow). Original magnification ×1,000.

Despite treatment for 5 days with clindamycin and quinine, the treatment of choice for severe babesiosis, the fever persisted. Antimicrobial drug therapy was changed to atovaquone and azithromycin. The patient completed 10 days of treatment and showed resolution of symptoms and normalization of platelet count and total bilirubin level. Parasitemia level at the time of discharge was 1.2%. Laboratory data after discharge were not available.

Patient 2 was an 84-year-old woman with microcytic anemia who was hospitalized on June 8, 2012, because of 2 weeks of fever, diaphoresis, myalgias, progressive dyspnea, and fatigue. Originally from New Hampshire, she had been living in Northampton County, Pennsylvania, for >4 years. She had not traveled outside the region in the past 4 years. She received a blood transfusion for chronic anemia 1 year before onset of this illness. She recalled multiple tick bites in the recent past.

Because of pancytopenia (Table), a bone marrow biopsy was performed and showed intraerythrocytic ring forms with tetrads. Parasitemia level for a peripheral blood smear was 1.4%. A PCR result for *B. microti* was positive.

**Table:** Clinical and laboratory data for 3 patients with babesiosis at dates of admission, diagnosis of babesiosis and discharge, eastern Pennsylvania, USA*

Characteristic	Patient 1		Patient 2			Patient 3	
Date	2011 Aug 18	2011 Aug 25	2012 Jun 8	2012 Jun 12	2012 Jun 16	2012 Jun 26	2012 Jul 11
Temperature, °C	38.7	35.7	36.8	37.2	36.1	37.8	37.2
Hematocrit, %	43.1	28.3	26.0	27.4	30.7	43.3	32.9
Leukocyte count, × 10^9^/L	9.87	6.43	2.94	5.47	3.36	7.93	10.6
Platelet count, × 10^9^/L	45	197	46	68	68	41	74
Total bilirubin, mg/dL†	4.77	0.88	3.05	NA	NA	2.0	2.48
Parasitemia level, %	10.0	1.2	NA	1.4	0.1	0.4	0.0
Creatinine, mg/dL‡	1.9	1.1	1.47	1.12	NA	1.16	0.95

*NA, not available. †Reference range 0.20–1.00 mg/dL. ‡Reference range 0.60–1.30 mg/dL.

The patient was given atovaquone and azithromycin for 10 days and showed resolution of symptoms and improvement in abnormal laboratory values. A repeat blood smear on June 18 showed a parasitemia level of 0.1%, and a blood smear 1 month later showed no parasites.

Patient 3 was a 71-year-old man who was hospitalized on June 26, 2012, because of 2 weeks of fever and gradually worsening malaise and weakness. He had not traveled outside eastern Pennsylvania in the past 2 years and never received a blood transfusion. Two weeks before admission, he had an insect bite that developed into a larger, oval rash.

Initial testing for thrombocytopenia included a peripheral blood smear, which showed intraerythrocytic ring forms and tetrads and a parasitemia level of 0.4%. A PCR result was positive for *B. microti*. He was given atovaquone and azithromycin for 10 days. Because results of enzyme immunoassay and Western blot for *B. burgdorferi* were positive, he was also given doxycycline. Symptoms resolved, and the laboratory values improved. A repeat blood smear 1 week after starting antimicrobial drug therapy showed no parasites.

## Conclusions

This report supports the hypothesis that babesiosis caused by *B. microti* is emerging in eastern Pennsylvania. As *B. microti* is spread by *I. scapularis* ticks, this infection might emerge in the range of the vector. Babesiosis is considered endemic to some states in which *I. scapularis* ticks are prevalent (*6*,*7*), but not in Pennsylvania.

All of these cases were confirmed by using robust laboratory methods. The 3 cases showed intraerythrocytic tetrad forms, an uncommon finding that is considered pathognomonic for *Babesia* infection (*1*). The strength of the diagnoses was further enhanced by PCR testing. Although PCR will amplify all *Babesia* species that are pathogenic in humans, the peak melting point temperature of amplicons for the 3 samples was nearly identical (difference ≤0.2°C) to that of the control *B. microti* amplicon, supporting *B. microti* as the causative agent (C.P. Cartwright, ViroMed Laboratories, Minnetonka, MN, USA, pers. comm.).

Two other persons with babesiosis have been treated at our institution since 2011. These case-patients were not included in this report because of inadequate laboratory confirmation for 1 patient and lack of a strict travel limitation for the other patient. One of these patients was given a diagnosis of babesiosis on the basis of serologic data obtained 2 months after the initial illness without blood smear confirmation. The second patient had traveled to western New Jersey, a babesiosis-endemic region, over a brief period before his illness.

The Pennsylvania Department of Health has received 39 voluntary reports of smear-positive babesiosis during 2005–2012 (K. Waller, Pennsylvania Department of Health, Harrisburg, PA, USA, pers. comm.). More than 60% of those reports were made after babesiosis became nationally reportable in 2011. Although many of these cases lacked detailed historical information to confirm that the cases were acquired in Pennsylvania, they further support the notion that *B. microti* is emerging in Pennsylvania.

Data on the prevalence of *B. microti* in *I. scapularis* ticks in eastern Pennsylvania are limited. However, 0.7% (3/443) of ticks collected from animals and humans in Monroe County in eastern Pennsylvania during 2004–2006 were infected with *B. microti* (*3*), supporting the plausibility of zoonotic transmission in the state.

Before these patients were identified, no cases of babesiosis had been diagnosed at our tertiary care center over the previous 10 years. Cases could have been missed because of the nonspecific nature of symptoms. However, this increase in the number of documented cases in 1 hospital is unlikely to be entirely explained by missed diagnoses. Furthermore, no other cases of babesiosis have been described at other institutions in this region.

This study had a few limitations. Recall bias could have played a role in obtaining a travel history. However, for each of these patients, family members were able to confirm the travel history, making recall bias unlikely. One of the patients had a blood transfusion 1 year before onset of systemic symptoms, making a transfusion-related infection unlikely. However, because this same patient was originally from New Hampshire, she might have been a chronic carrier.

These cases support the premise that babesiosis caused by *B. microti* infection is emerging in eastern Pennsylvania. Knowledge of the geographic distribution of *B. microti* is essential. Diagnosis requires strong clinical suspicion and supportive laboratory data. Timely diagnosis and treatment for patients and testing of blood donors in areas in which *B. microti* is found might further prevent transfusion-related infection (*8*–*10*). Having this infection reportable in Pennsylvania and other states to which *I. scapularis* ticks are endemic might help identify the geographic region of this parasite.
